# Diet Is Critical for Prolonged Glycemic Control after Short-Term Insulin Treatment in High-Fat Diet-Induced Type 2 Diabetic Male Mice

**DOI:** 10.1371/journal.pone.0117556

**Published:** 2015-01-29

**Authors:** Aili Guo, Nigel A. Daniels, Jean Thuma, Kelly D. McCall, Ramiro Malgor, Frank L. Schwartz

**Affiliations:** 1 Ohio University Heritage College of Osteopathic Medicine, Athens, Ohio 45701, United States of America; 2 The Diabetes Institute at Ohio University, Athens, Ohio 45701, United States of America; Hochschule Bremen, GERMANY

## Abstract

**Background:**

Clinical studies suggest that short-term insulin treatment in new-onset type 2 diabetes (T2DM) can promote prolonged glycemic control. The purpose of this study was to establish an animal model to examine such a “legacy” effect of early insulin therapy (EIT) in long-term glycemic control in new-onset T2DM. The objective of the study was to investigate the role of diet following onset of diabetes in the favorable outcomes of EIT.

**Methodology:**

As such, C57BL6/J male mice were fed a high-fat diet (HFD) for 21 weeks to induce diabetes and then received 4 weeks of daily insulin glargine or sham subcutaneous injections. Subsequently, mice were either kept on the HFD or switched to a low-fat diet (LFD) for 4 additional weeks.

**Principal Findings:**

Mice fed a HFD gained significant fat mass and displayed increased leptin levels, increasing insulin resistance (poor HOMA-IR) and worse glucose tolerance test (GTT) performance in comparison to mice fed a LFD, as expected. Insulin-treated diabetic mice but maintained on the HFD demonstrated even greater weight gain and insulin resistance compared to sham-treated mice. However, insulin-treated mice switched to the LFD exhibited a better HOMA-IR compared to those mice left on a HFD. Further, between the insulin-treated and sham control mice, in spite of similar HOMA-IR values, the insulin-treated mice switched to a LFD following insulin therapy did demonstrate significantly better HOMA-B% values than sham control and insulin-treated HFD mice.

**Conclusion/Interpretation:**

Early insulin treatment in HFD-induced T2DM in C57BL6/J mice was only beneficial in animals that were switched to a LFD after insulin treatment which may explain why a similar legacy effect in humans is achieved clinically in only a portion of cases studied, emphasizing a vital role for diet adherence in diabetes control.

## Introduction

Type 2 diabetes mellitus (T2DM) is characterized by visceral obesity-induced insulin resistance and progressive deterioration of pancreatic β-cell function [1–3]. Most of the microvascular complications of diabetes are related to the degree and the length of exposure to hyperglycemia. Data from the follow-up study of the United Kingdom Prospective Diabetes Study (UKPDS) emphasizes the role of glycemic control early in the course of the disorder and its value in delay or prevention of later complications. The phenomenon of prolonged beneficial effects on diabetic complications after a period of improved glycemic control, regardless of subsequent treatments and degree of glycemic control, has been described as a “legacy effect”. Such a concept of a “legacy effect” or “metabolic memory” may also be extended to the favorable outcomes observed in diabetic “remission” after a short-term, early insulin treatment (EIT) in new-onset T2DM. For instance, at two-year follow-up after a short course of intensive insulin therapy in new-onset T2DM, 42–69% of patients remained free of hyperglycemia [4, 5].

It is believed that EIT improves multiple adverse metabolic consequences by rapidly overcoming “glucolipotoxicity” in β-cells and other insulin target cells; especially liver and muscle, thereby reducing endogenous insulin requirements, facilitating “β-cell rest”, protecting β-cell function, and by improving insulin sensitivity [6, 7]. Insulin also possesses anti-inflammatory and antioxidant properties that may help protect against endothelial dysfunction and vascular complications [8–11], positively altering the course of vascular disease progression. However, many gaps in our knowledge remain in this area; in particular, why is this legacy effect of EIT in glycemic control only observed in a portion of patients described in clinical studies? Furthermore, the role of diet adherence in the prolonged diabetic control following EIT is not usually analyzed. The present study aimed to study the legacy effects of EIT on a high-fat diet (HFD)-induced obese T2DM mouse model. Specifically, it was designed to characterize the role of diet on the favorable outcomes of EIT in new-onset T2DM. We demonstrate that switching to a LFD following EIT is critical to attaining the legacy benefit from insulin therapy. In addition, the anti-inflammatory effect of EIT on islet inflammation was also evaluated.

## Materials and Methods

### Animals and Insulin Treatment

**Fig 1:**
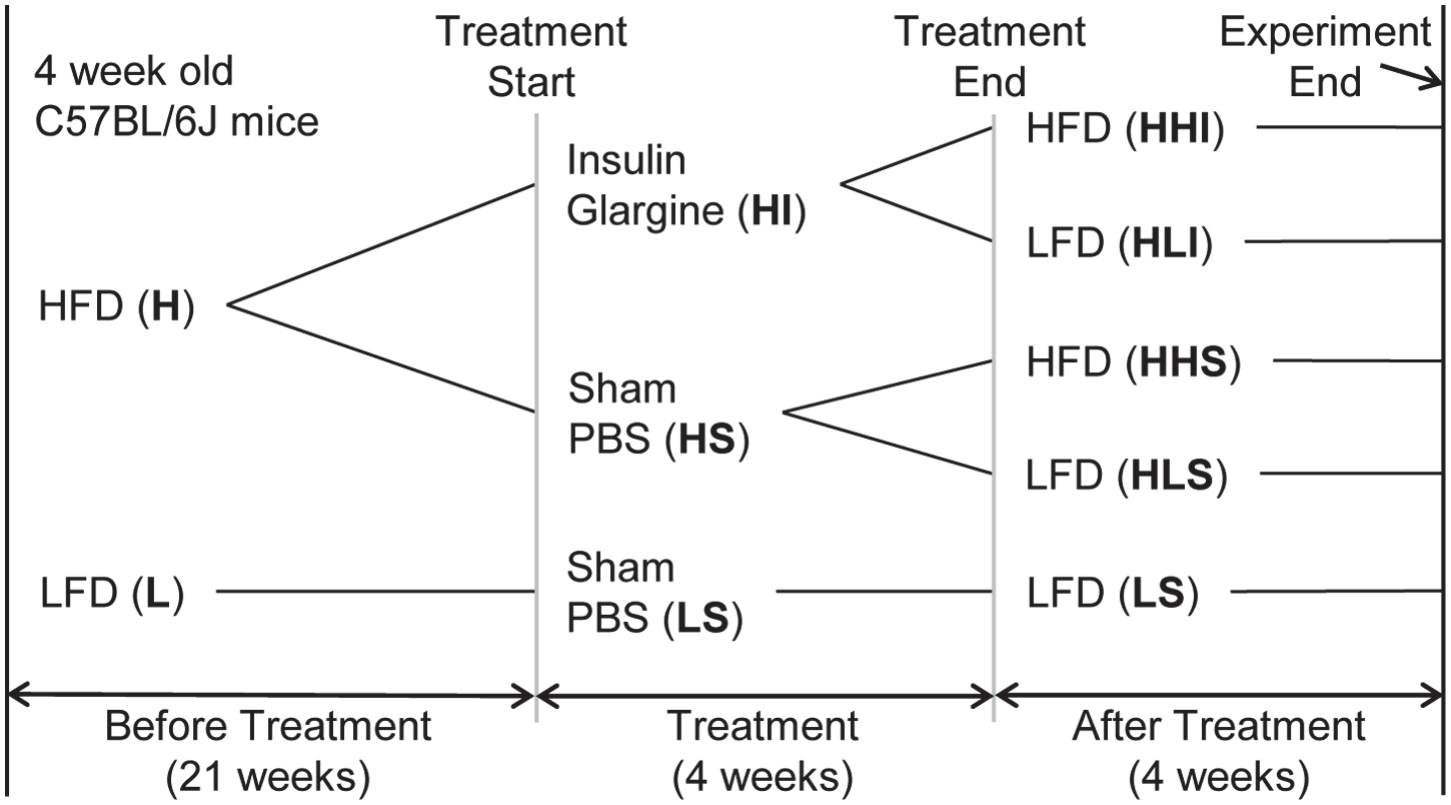
Schematic design of experiments. 4 week-old mice were fed a HFD (H) or a LFD (L) for 21 weeks and then received either daily subcutaneously-injected insulin Glargine (HI) or sham phosphate-buffered saline (HS) for 4 weeks. Mice were then kept on a HFD (HHI or HHS) or switched to a LFD (HLI or HLS) for 4 more weeks before being killed. Another group of mice fed a LFD throughout the study received sham treatment and served as controls (LS). A schematic diagram illustrating the different treatment groups is shown in Fig. 1.

Four week-old C56BL6/J male mice were purchased from the Jackson Laboratory, Bar Harbor, ME, USA. Animals were housed in a restricted access facility in groups of 2–4, with a constant room temperature of 24°C and a 14 hour light/10 hour dark cycle and an access to food and water ad libitum. Mice were placed on a HFD (60% kcal from fat, Research Diets, INC., New Brunswick, NJ, USA), and after 21 weeks, an intraperitoneal glucose tolerance test (GTT) was performed to confirm their diabetic status. Mice were then randomly placed into treatment groups and began to receive either daily subcutaneous insulin glargine (Lantus, Sanofi-Aventis U.S. LLC, Bridgewater, NJ, USA) or sham phosphate-buffered saline (PBS) for four weeks at 9 AM in their home cage in a procedure room. A safe and effective insulin dosage to target day time non-fasting glucose levels of 5.33–8.33 mmol/L (100–150 mg%) without causing significant hypoglycemia was determined by a pilot study according to the literature [12]. Proper glycemic control was achieved by titrating up the insulin dose within a week and average insulin daily dosage was 4–7 IU per mouse (see S1 Fig. for non-fasting blood glucose levels). Mice were then kept on a HFD or switched to a chow diet, i.e., low-fat diet (LFD, 10% kcal from fat, ProLab RMH3000 5P00, LabDiet, St. Louis, MO, USA) for four more weeks before being killed by exsanguination under tribromoethanol anesthesia. Another group of mice fed a LFD throughout the study served as controls. A schematic diagram illustrating the different treatment groups is shown in Fig. 1. At the end of experiment, each study group contains 7–18 mice. All animal experiments were performed according to the protocols approved by the Ohio University Institutional Animal Care and Use Committee (IACUC Protocol Number# 12-H-032), Athens, Ohio.

### Body weight and body composition analysis

Body weight measurements were conducted every week at 9 AM in their home cage in a procedure room. Body composition measurements were performed every four weeks in the afternoon in their home cage moved to a laboratory and using a Minispec mq benchtop nuclear magnetic resonance analyzer (Bruker Instruments, Billerica, MA, USA) as previously described [13, 14].

### Measurement of fasting blood glucose, insulin, C-peptide, leptin, and cholesterols

Fasting blood glucose and hormones were measured before starting insulin treatment and at the end of the experimental period in the same mice analyzed for body composition. One day after body composition measurements, mice were fasted for six hours, and blood was collected from the tail tip within a 2 hour time frame starting at 2 PM. Fasting blood glucose levels were determined using the first drop of blood collected from the tip of the tail to minimize stress-induced changes in glucose levels by using a OneTouch UltraMini glucometer and OneTouch Ultra Blue test strips (LifeScan, Inc., Milpitas, CA, USA). Blood was centrifuged at 4°C to isolate the plasma, which was then stored at −80°C until future use. Blood levels of insulin, c-peptide, and leptin were determined using a MILLIPLEX MAP multiplex assay (EMD Millipore Corporation, Billerica, MA, USA). The intra-assay coefficients of multiple determination for insulin, c-peptide, and leptin measurements were R^2^ = 0.999, 0.999 and 0.997, respectively. Blood cholesterol and triglyceride levels were measured using a Cholestech LDX Analyzer (Alere Inc., Waltham, MA, USA); and the intra-assay coefficients of variation for cholesterol and triglycerides measurements were 5% and 7%, respectively.

### The Homeostasis Model Assessment (HOMA) analysis

HOMA, published in 1985 [15] and further modified in 1998 [16], has been widely used to estimate steady state β-cell function (HOMA-B) and insulin resistance (HOMA-IR) in multiple animal studies including those using HFD-induced obesity and impaired glucose intolerance [17–19]. We have calculated HOMA-IR and HOMA-B based on the following formulae: HOMA-IR = (Fasting Glucose x Fasting Insulin)/22.5 and HOMA-B = (20 x Fasting Insulin)/ (Fasting Glucose-3.5) %.

### Glucose tolerance test (GTT) and Insulin tolerance test (ITT)

Mice were fasted in clean cages with fresh corn cob bedding for six hours in their housing room before being moved to a procedure room for the GTT that began at 2 PM. 10% glucose in PBS was intraperitoneally (IP) administered at a dose of 1 mg glucose per gram body weight. Glucose levels were measured by using OneTouch UltraMini glucometers (LifeScan) at 0, 5, 15, 30, 60, 90, and 120 minutes. GTTs were performed before and after 4 weeks of treatment as well as at the end of the experimental period. At the end of the experimental period, an ITT was performed on each group of animals. Non-fasted mice weighed and baseline blood glucose measurements were obtained. Insulin was administered to each mouse via IP injection at a dosage of 0.75 units/kg of body weight. Blood glucose measurements were then taken 15, 30, 60, 90 and 120 minutes after insulin administration.

### Islet Immunohistochemistry

Insulin staining was performed on formalin-fixed paraffin-embedded pancreatic tissue sections at the end of the experiment. Briefly, after deparaffinization, sections were washed in PBS buffer and incubated overnight at 4°C with antiserum raised in rabbit against mouse insulin (Abcam, Cambridge, MA, USA). Sections were then treated using an EXPOSE Rabbit-specific HRP/DAB detection IHC kit according to the manufacturer’s instructions (Abcam, Cambridge, MA, USA). The specificity of the antiserum was tested by comparison with isotype control antibody staining using Rabbit polyclonal IgG (Abcam, Cambridge, MA, USA) under identical conditions. Staining was visualized using a Nikon Microphot-SA microscope (Nikon Inc., Melville, NY, USA), captured using a MicroPublisher 5.0 RTV camera (QImaging, Surrey, BC, Canada) and analyzed using Image-Pro Plus software (Media Cybernetics, Inc., Rockville, MD, USA). To evaluate the difference in the total number of pancreatic islets between treatment groups, islets were counted under the microscope in 10 different fields at 20x magnification. Slides stained for insulin were evaluated for the presence of peri-insulitis, which was determined as the percentage of islets showing the presence of mononuclear cell masses within or immediately adjacent to the islet. The number of islets in each group was calculated, followed by the average number of islets per field. Islet size was defined as follows: small islet: 1–5 cross-sectional cells, medium islet: 5–20 cross-sectional cells, large islet: 20–50 cross-sectional cells [20].

### Apoptotic assay of pancreatic islets

At the end of the experimental period, apoptosis of pancreatic tissues was assessed using a terminal deoxynucleotidyl transferase dUTP nick end labeling (TUNEL) assay (DeadEnd colorimetric TUNEL kit, Promega, Madison, WI, USA) as described by the manufacturer. The numbers of TUNEL-positive cells/islet were counted under microscope in 10 different fields at 20x magnification.

### Statistical analysis

Data were collected from all animals used, and are expressed as Mean ± SE. Data of repeatedly measured variables (i.e., body weight, body composition, hormone levels, and blood glucose) were subjected to two-way ANOVAs along with *t*-tests for select comparisons. Results were considered significant if p < 0.05. To analyze TUNEL-positive cells of pancreatic tissues, a multi-level modeling was used, i.e., the fields nested within the slides nested within animals. In this analysis slices and fields will be treated as level-2 and level-3 random factors, respectably, and the difference between treatment groups was tested.

## Results

### Mice fed a HFD became heavier and fatter

**Fig 2:**
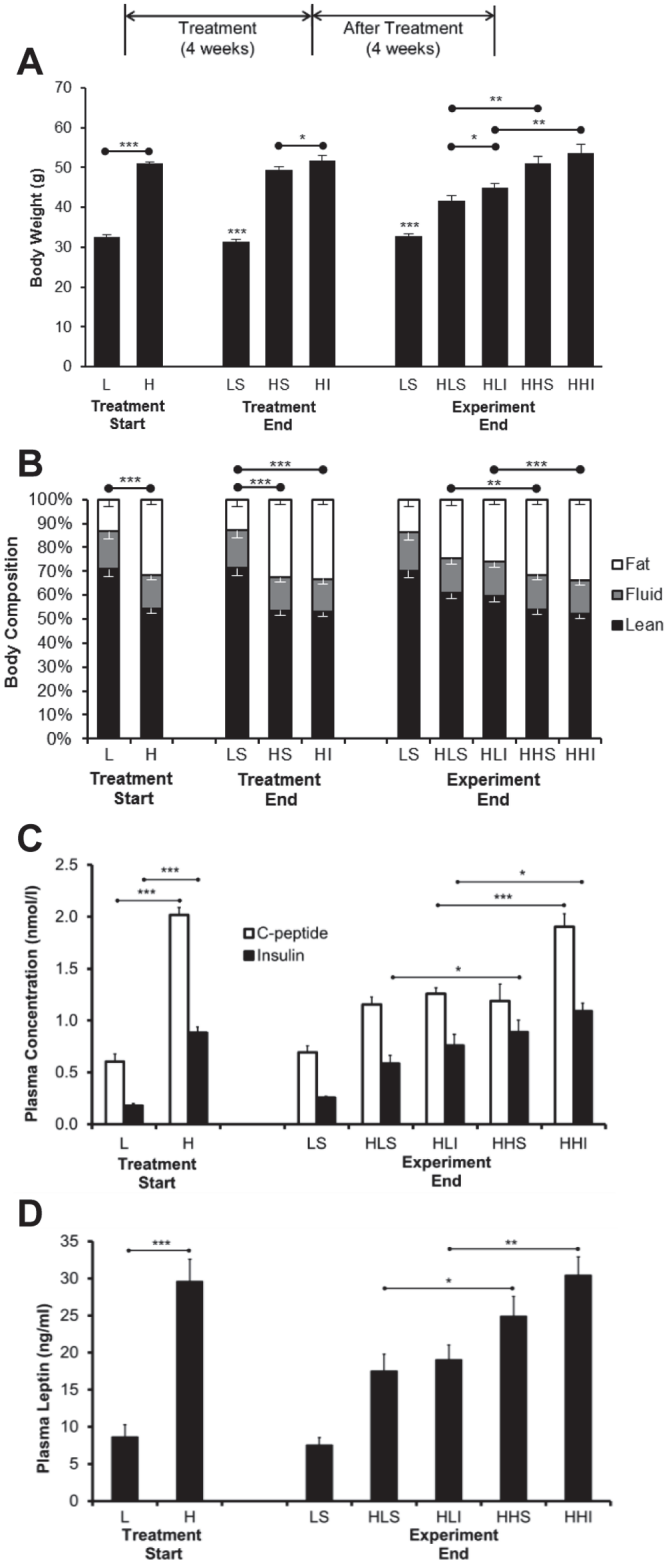
Changes in body weight, composition, blood C-peptide/insulin, and Leptin levels in different groups of mice (A) Mice fed a HFD gained significant body weight when compared to that of LFD mice (***p<0.001, H vs. L). After the treatment period, insulin-treated HFD mice were significantly heavier, when compared to sham-treated HFD mice (*p<0.05, HI vs. HS). At the end of the experiment, mice that were switched to a LFD were lighter than HFD mice (**p<0.01, HLS vs. HHS and HLI vs. HHI). (B) Body composition was determined by a benchtop nuclear magnetic resonance analyzer. Before initiating and after terminating treatment, mice fed a HFD were fatter (fat mass in white), compared with that of LFD fed mice (***p<0.001, L vs. H and LS vs. HS and HI). At the end of the experiment, mice that switched to the LFD lost more fat mass, when compared to mice kept on a HFD (***p<0.001, HLI vs. HHI; **p<0.01 HLS vs. HHS). (C) Prior to treatment, blood C-peptide/insulin levels were significantly greater in HFD fed mice, in comparison to that in LFD controls (***p<0.001, H vs. L). In the end of experimental period, Sham treated control mice that were kept on HFD displayed increased plasma insulin levels than those switched to LFD after treatment (*p<0.05, HLS vs. HHS) (n = 6–8 per group). (D) Prior to treatment, mice fed a HFD demonstrated higher blood levels of leptin than LFD fed mice (***p<0.001, H vs. L). At the end of the experiment, mice that were kept on the HFD continued to show high levels of leptin compared to mice switched to the LFD regardless of therapy (**p<0.01, HHI vs. HLI; *p<0.05, HHS vs. HLS).

Mice fed a HFD for 21 weeks compared to LFDT animals developed fasting hyperglycemia and elevated HbA1c indicating the induction of T2DM (Table 1). Animals on the HFD also gained much more weight, exhibited higher total body fat content (Fig. 2A & B), as well as higher fasting insulin, C-Peptide, and leptin levels (2C & D) compared to mice fed a LFD. During the 4 week intervention period following the onset of diabetes, the insulin-treated mice maintained on a HFD continued to gain weight, more body fat (Fig. 2A & B), and even higher fasting insulin, C-Peptide, and leptin levels (2C & D) whereas sham-treated diabetic mice showed a tendency to lose weight (Fig. 2A). However, in spite of the weight loss seen in untreated mice, the body compositions in each group, i.e., percentage of lean, fat and fluid content, were not different (Fig. 2B). After the insulin treatment phase, diabetic mice maintained on the HFD continued to gain weight while mice switched to a LFD lost weight similar to the sham-treated animals (Fig. 2A & B). The diabetic mice maintained on the HFD also demonstrated significantly also higher fasting insulin, C-peptide, and leptin levels in comparison to insulin-treated LFD-fed mice and sham controls (Fig. 2C & D).

**Table 1:** Fasting blood glucose and HbA1c of C56BL6/J male mice after fed a LFD (L) or a HFD (H) for 21 weeks.

Group	Fasting Blood Glucose (mmol/L)	HbA1c (mmol/mol)
**L**	10.70 ± 0.47 (n = 24)	26.91 ± 0.54 (n = 16)
**H**	13.19 ± 0.23 (n = 90)	31.92 ± 0.52 (n = 74)
*p value*	<0.0001	<0.0001

L, mice fed a LFD. H, mice fed a HFD.

A similar pattern of higher total blood cholesterol levels was observed in the various treatment groups while triglyceride concentrations were not significantly different in any group (S2 Fig.).

### Insulin therapy resulted in better glycemic control and improved GTT performance

**Fig 3:**
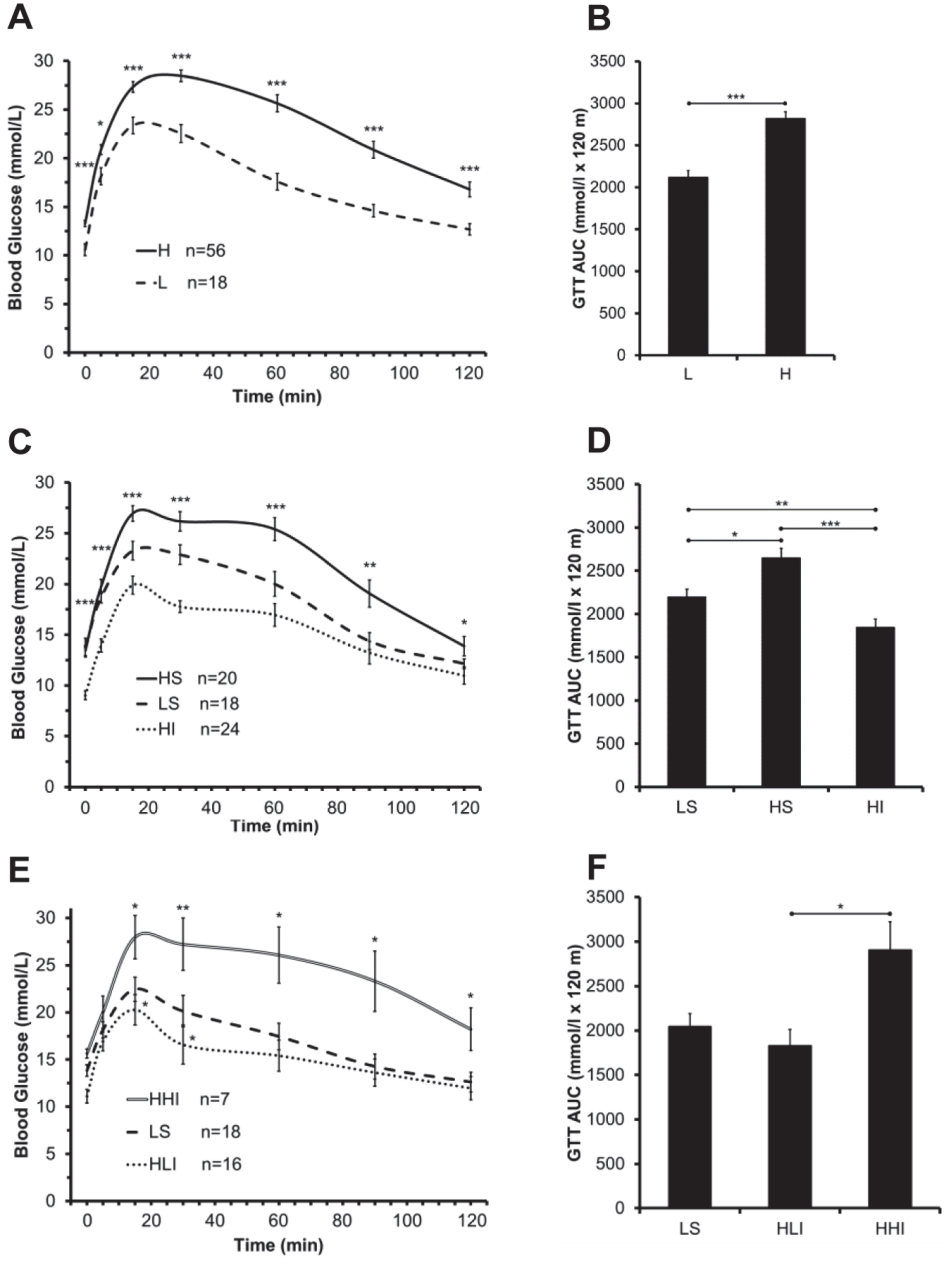
Effect of short-term insulin therapy on glycemic control. (A) and (B) Mice fed a HFD showed worse GTT performance, when compared to that of mice fed a LFD (***p<0.001, and *p<0.05, H vs. L). (C) and (D) Insulin therapy significantly enhanced GTT performance in HFD mice, in comparison to that of sham controls (***p<0.001, **p<0.01, and *p<0.05, HI vs. HS). (E) and (F) At the end of the experiment, insulin-treated mice that switched to a LFD after treatment demonstrated significantly better GTT performance, in comparison to that of sham controls (*p<0.05, HLI vs. HLS at 15 and 30 min). Conversely, insulin-treated mice that were kept on a HFD until the end of the experiment exhibited much worse GTT performance, in comparison to that of mice switched to the LFD (**p<0.01, and *p<0.05, HHI vs. HLI).

As expected, mice fed a HFD for 21-weeks demonstrated hyperglycemia at baseline (blood glucose levels: 13.27 ± 0.31 vs. 10.57 ± 0.60 mmol/L, mean ± SE, *p<0*.*001*), impaired GTT performance and much higher area under curve (AUC)’s when compared to mice fed a LFD (Fig. 3A & B). Four weeks of insulin therapy in mice maintained on the HFD demonstrated significantly improved GTTs compared to no insulin (sham treated) animals with lower glucose levels and AUC following IP glucose challenge (Fig. 3C &D). After the insulin treatment was discontinued, the diabetic mice switched to a LFD lost weight (Fig. 2A) which were similar to the losses observed in the sham-treated animals and demonstrated significantly better GTT performance and AUC in comparison to sham controls or mice maintained on the HFD (Fig. 3E & F). Mice remaining on a HFD following cessation of the insulin treatment exhibited no benefit from the early insulin therapy and demonstrated even worse GTT performance and much higher AUC’s at the end of the experimental period compared to sham-treated and insulin-treated diabetic mice (Fig. 3E & F).

### Early insulin-treated mice improved HOMA-B%

**Fig 4:**
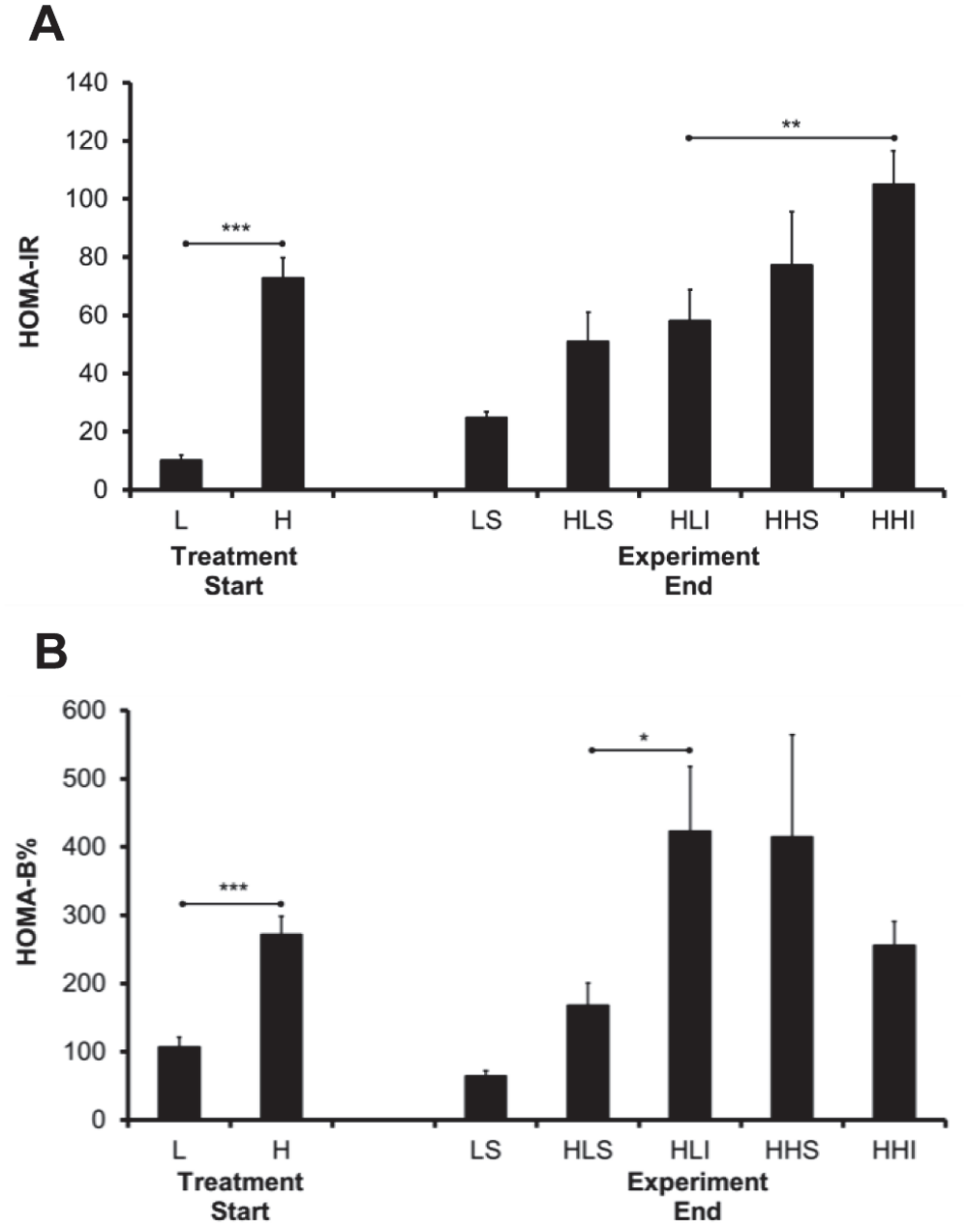
Changes in HOMA-IR and HOMA-B values after insulin therapy. Changes in HOMA-IR and HOMA-B values after insulin therapy. Prior to treatment, HOMA-IR values (A)and HOMA-B (B) were significantly greater in HFD fed mice, in comparison to that in LFD controls (***p<0.001, H vs. L). At the end of the experiment, the insulin-treated mice that were kept on the HFD after treatment displayed worse HOMA-IR levels (A) in comparison to mice switched to the LFD after treatment (***p<0.001, **p<0.01, *p<0.05, HHI vs. HLI). Moreover, the latter demonstrated a significantly improved HOMA-B value than that of sham controls (*p<0.05, HLI vs. HLS) despite similar HOMA-IR values.

The 21 week-HFD feeding of mice, as expected, resulted in much higher HOMA-IR and HOMA-B% compared to LFD (Fig. 4A &B) as insulin resistance paralleled increases in body weight, body fat, fasting insulin, C-peptide levels, at the onset of diabetes. As seen with GTT performance and AUC studies (Fig. 3C & D), early insulin-treated mice maintained on the HFD also exhibited the highest HOMA-IR values of any group (Fig. 4A).Insulin-treated mice switched to the LFD exhibited a better HOMA-IR compared to those mice left on a HFD (Fig. 4A). In spite of similar HOMA-IR values, between the insulin-treated and sham control mice, the insulin-treated mice switched to a LFD following insulin therapy did demonstrate significantly better HOMA-B% values than sham control and insulin-treated HFD mice (Fig. 4B). There was no significant difference in ITT results among animal maintained on the HFD (HLS, HLI, HHS and HHI) groups (S1 Table).

### Greater numbers of large pancreatic islets were seen in HFD-fed mice irrespective of insulin treatment

**Fig 5:**
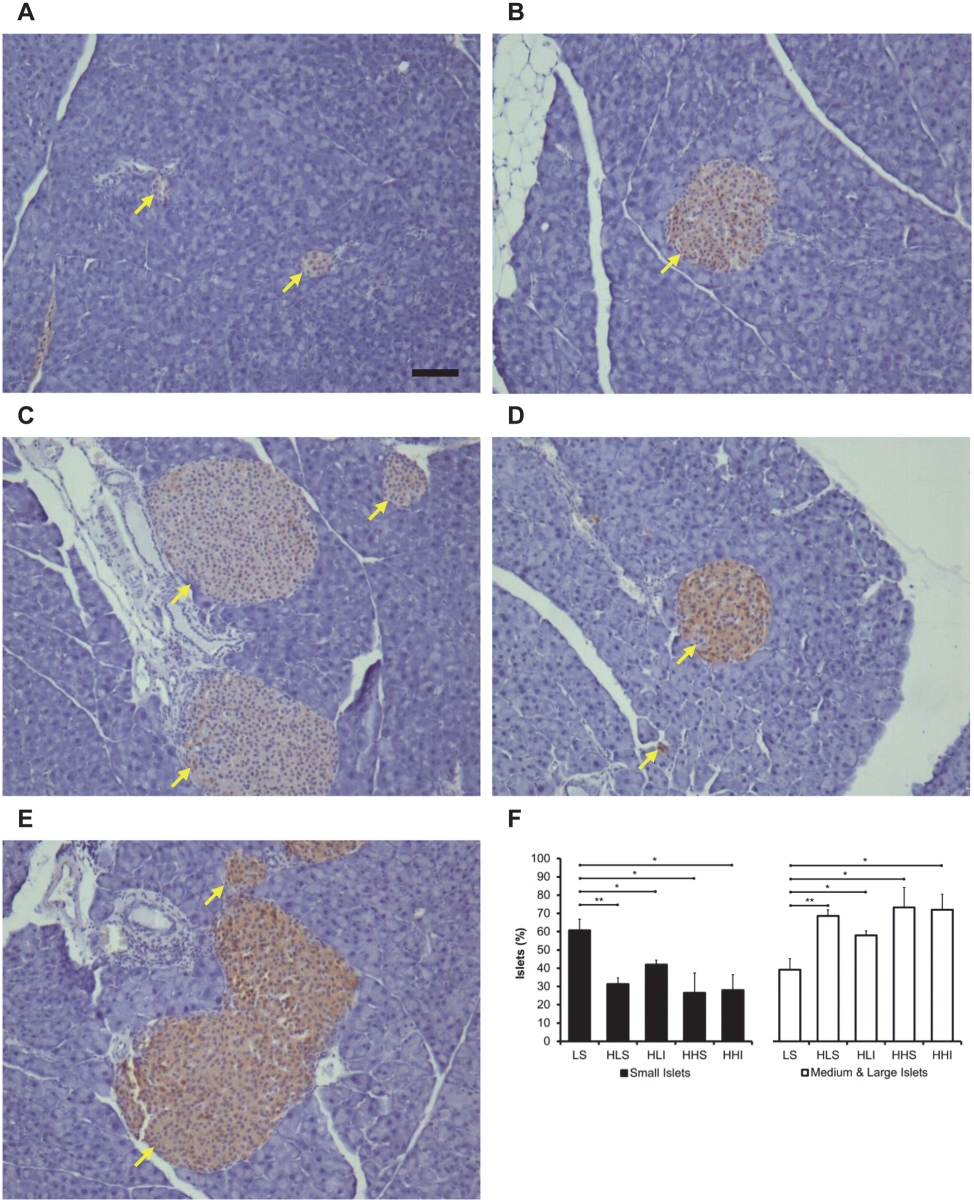
Effect of diets and insulin treatment on the number and size of pancreatic islets in mice. (A) to (F) At the end of the experimental period, mice fed the LFD exhibited predominantly small-sized islets (**p<0.01 LS vs. HLS; *p<0.05, LS vs. HLI, HHS, HHI). Conversely, HFD fed mice showed more medium- and large-sized islets (**p<0.01 LS vs. HLS; *p<0.05, LS vs. HLI, HHS, HHI), which, however, were not significantly different among treatment groups (n = 4–8 per group). Scale bar = 100 μm.

Mice maintained on the LFD throughout the experiment exhibited predominantly small-sized pancreatic islets regardless of insulin therapy at onset of diabetes (Fig. 5). Insulin treated mice who were switched to the LFD appeared to have more small-sized islets at 4 weeks compared the mice maintained on the HFD (Fig. 5).

### TUNEL-positive islets were detected in HFD fed mice, but unaffected by insulin treatment

**Fig 6:**
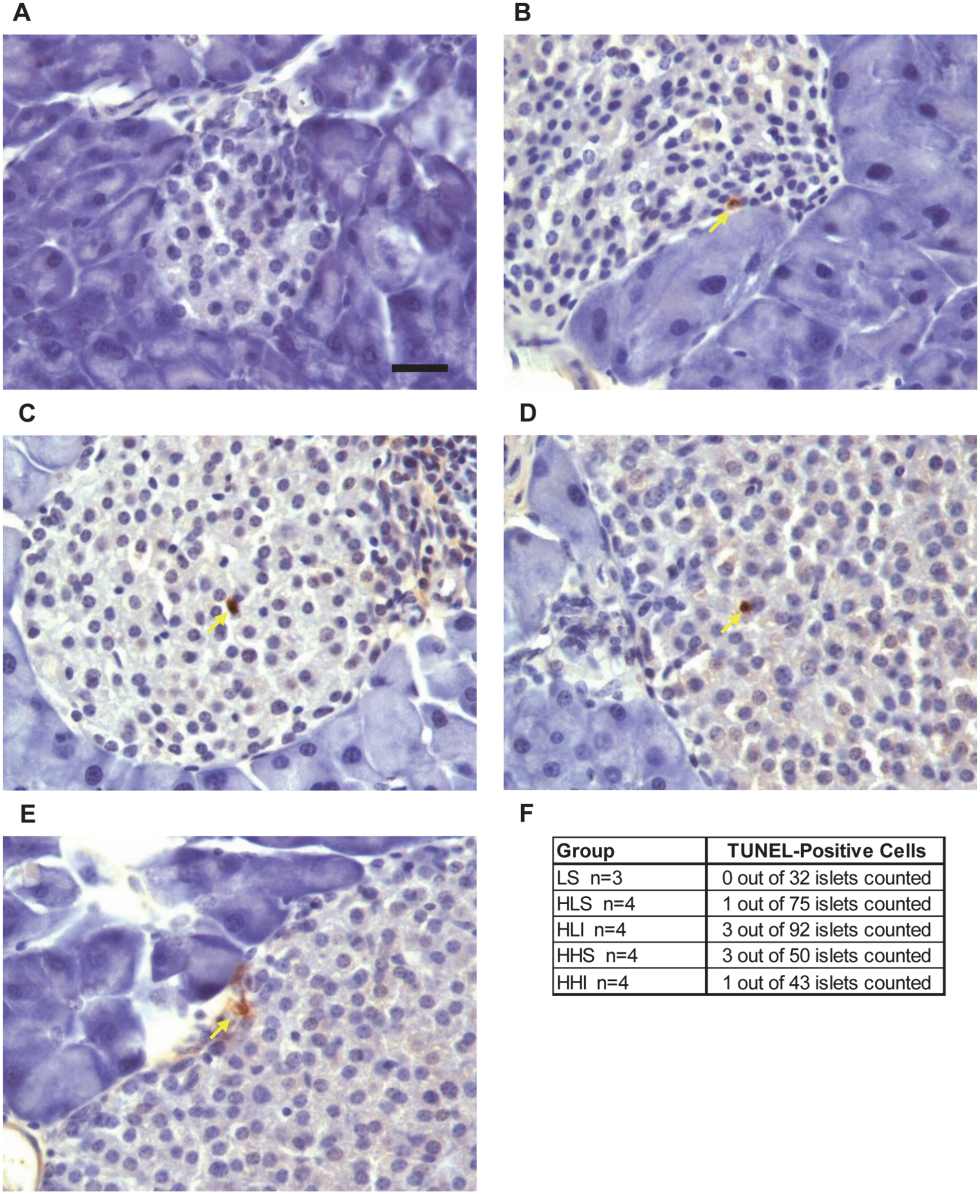
Effect of insulin therapy on islet inflammation and apoptosis. Whilst no apoptosis was identified in islets from LFD controls (A), TUNEL-positive cells were found in pancreatic tissues from HFD fed mice (B-E), but were not different between treatment groups (F). Of note, special attention was paid to assess peri-insulitis and none was identified. Scale bar = 10 μm.

To see if the HFD lead to apoptosis of β-cells TUNEL assay was assessed in all treatment groups (Fig. 6). No TUNEL-positive cells were detected in pancreatic tissues from LFD. Scattered TUNEL-positive cells were detected in pancreatic tissues from HFD mice but no significant effect of insulin treatment was noted (Fig. 6). Special attention was also paid to assess evidence for possible peri-insulitis being triggered by the HFD or onset of diabetes and none was identified.

## Discussion

The natural history of T2DM is a progression of β-cell failure and loss of β-cell mass over time. Reversal of β-cell dysfunction and prevention of β-cell loss have become a major goal of therapy. Recent clinical studies have suggested that intensive EIT in newly diagnosed T2DM holds promise as a potential disease-modifying therapeutic option with significant durability [7, 21–26]. However, the mechanism(s) by which EIT contributes to this legacy effect remains to be elucidated. For instance, following a short course of intensive insulin therapy in newly diagnosed T2DM, some patients had favorable long-term remission but others do not. As suggested by a recent meta-analysis [27], the proportion of participants in drug-free remission after EIT was about 66% after 3 months, about 59% after 6 months, and 46% after 12 months, and about 42% after 24 months of follow-up. Because information about diet adherence is often lacking in clinical studies, how diet control affects the outcomes of such a beneficial effect of EIT is unknown. We have used a high-fat diet animal model to mimic common human dietary habits to study the impact of diet on the potential beneficial effect of early, short-term, insulin therapy in new-onset T2DM.

The present animal study shows that a four-week course of insulin therapy significantly improved GTT performance in new-onset HFD-induced T2DM mice. However, EIT only conferred a beneficial effect in animals switched to a LFD after the insulin treatment. Insulin-treated mice switched to the LFD displayed a better HOMA-IR as expected compared to those mice left on a HFD. Further, between the insulin-treated and sham control mice, in spite of similar HOMA-IR values, the insulin-treated mice switched to a LFD following insulin therapy did demonstrate significantly better HOMA-B% values than sham control and insulin-treated HFD mice. Thus, EIT, coupled with a LFD after insulin treatment, resulted in weight loss, reduced leptin levels, improved GTT performance and a better HOMA-B% compared to sham untreated controls and to EIT in mice maintained on the HFD. In contrast, maintaining a HFD of those insulin-treated diabetic mice overwhelms any potential benefit of EIT by promoting weight gain and worsening glucolipotoxicity and insulin resistance. Taken together, our findings, on one hand, emphasize the fundamental role of diet compliance to reach proper glycemic control in any kind of diabetic regimen; and, on the other hand, demonstrate that the legacy effect of EIT observed in clinical studies was only observed in diabetic mice switched to a LFD. The current study is the first report of beneficial effect of glycemic control after cessation of insulin therapy in an animal model of T2DM with specific emphasis on the role of diet control in the legacy effect of EIT. Retrospectively, we should have initiated the LFD at onset of diabetes and at the initiation of EIT which we predict would enhance and extend the legacy effect.

Previous studies have suggested that intensive EIT confers sustained normoglycemia beyond the short-term treatment period in new-onset T2DM with improved β-cell function and insulin resistance. Li Y et al [7] reported that two-week intensive insulin therapy led to improvement in β-cell function, and restoration of first-phase insulin response, with many individuals achieving remission. Similarly, Son JW et al [6] showed that adequate glycemic control following 12-week insulin therapy was associated with restoration of β-cell function by showing improved insulin secretion, HOMA-B, and insulinogenic index. Likewise, a recent meta-analysis of clinical studies concluded beneficial effects from EIT in T2DM and tried to explain the mechanism of these beneficial effects, but did not contribute much to the mechanistic understanding of this effect [27]. The pooled analysis of seven studies (n = 839 participants) between 2004 and 2012 suggested that EIT improves β-cell function and insulin resistance in patients with early T2DM; specifically, a post-short-term intensive insulin therapy increase in HOMA-B as compared with baseline (1.13, 95% CI 1.02–1.25) and a decrease in HOMA-IR (-0.57, -0.84 to -0.29). In line with the clinical studies, our animal study demonstrated that EIT improved insulin sensitivity (HOMA-IR) but only in animals switched to the LFD at the end of experimental period. EIT plus switching to LFD also resulted in weight loss, improved glycemic control (GTT and AUC), as well as enhanced β-cell function as demonstrated by the favorable HOMA-B% by the end of experimental period. In contrast, although glucose control was improved with insulin in mice with diabetes while on the HFD, once the insulin was discontinued, glucose control worsened and β-cell functions (HOMA-B %) continued to deteriorate. It is worthwhile to mention that, the effect of the HFD on diabetes progression is initiated by insulin resistance rather than insulin deficiency in the animal model. As similar to clinical practice in treating patients with T2DM, insulin treatment per se in this animal study would not improve insulin resistance, rather likely induce more; especially during the treatment period. The critical role of a LFD in the legacy effect of EIT demonstrated in our study in mice raises an important issue as to how much dietary non-compliance affects the outcomes of published clinical EIT studies and these findings needs to be addressed in future clinical and animal EIT work.

Experimental HFD-induced obesity and impaired glucose homeostasis closely mimic clinical T2DM. It is well-known that increased fat, particularly visceral fat, is associated with proinflammatory status, insulin resistance, and β-cell dysfunction. In HFD-induced obesity, both acute tissue lipid overload and macrophage-induced proinflammatory actions mediate insulin resistance [28]. In the present study, mice fed a HFD became be obese and diabetic, as expected. Diabetic mice treated with EIT but maintained on the HFD demonstrated even greater weight gain and insulin resistance compared to sham-treated mice. As a compensatory response to obesity and insulin resistance, more medium- and large-sized islets were seen in those mice, which are similar to findings in type 2 diabetic patients [20, 29]. EIT had no effect on the size or numbers of islets regardless of diet although HOMA-IR and HOMA-B% improved in the animals switched to the LFD. Perhaps 4 weeks is too short of an observation interval to look at the potential β-cell protective effects of EIT. In addition, switching to a LFD upon onset of diabetes and initiation of EIT may show an earlier potential β-cell protective effect and future studies should investigate on this. It is noteworthy that during the four-week active insulin therapy period of the study, sham control mice lost body weight, possibly due to the catabolic state of the diabetes (and possibly injection-related stresses), while the insulin-treated mice were still on the HFD and gained a slight amount of weight. Insulin may cause weight gain through various mechanisms. Insulin is an anabolic hormone and causes muscle protein synthesis and lipogenesis [30]. It has a direct effect on sodium reabsorption in the renal distal tubule, causing accumulation of sodium and subsequent water retention [31]. Treatment-associated weight gain in diabetic patients is also related to a decrease in energy loss via glycosuria [32]. Nevertheless, the long-term benefits of tight glycemic control far outweigh the minor risks associated with this insulin treatment.

The justification for EIT is based on evidence demonstrating a multilayered benefit of insulin treatment. It has the ability to ameliorate glucotoxicity, to facilitate “β-cell rest”, and to promote proinsulin maturation and insulin secretion by attenuating endoplasmic reticulum-crowding, to possibly affect β-cell growth and survival via its anti-islet inflammatory effects [8, 9]. Besides, it also exerts beneficial effects on cardiovascular risk factors such as lipid profile, endothelial function, vasodilatation, and fibrinolytic profiles [8–11]. Recently, Li HQ et al [33] has shown that ten weeks of insulin treatment increased β-cell proliferation and β-cell area in glucose-intolerant rats while inhibiting pancreatic lipid accumulation and islet β-cell apoptosis.We did look for accelerated β-cell apoptosis by using TUNEL assay, which showed minimal increases in apoptotic changes in pancreatic tissue all animals on the HFD with no benefit from insulin which is similar to other studies; however, we did not test it during or immediately at the conclusion of the four week insulin therapy. We also tried to determine the effect of the HFD in triggering acute inflammation in the islets of mice of new onset T2DM. This was attempted based on the work by Ahrén group who reported extensive pancreatic inflammation and peri-insulitis in female mice of advanced age of 10 month old chronically fed a HFD for 11 months [34]. Our studies failed to demonstrate signs of peri-insulitis or accelerated apoptosis in new-onset T2DM, which had also been reported by the same group in young mice after one year of HFD feeding [35]. Pancreatic lipotoxicity as a mechanism was examined by using Oil Red O staining, but we found no difference among treatment groups. Also, circulating levels of multiple serum inflammatory cytokines (TNFα, IL-6, MCP-1) measured by Milliplex assay were not different among the groups (S2 Table). All these negative results are however in line with recently published studies showing that dedifferentiation of β-cells, rather than an apoptotic process appears to be the main mechanism behind β-cell mass loss in T2DM, and insulin therapy could reverse the process and allow β-cell redifferentiation [36, 37].

The first limitation of this study is that we did not determine the long-term durability of EIT on glucose control as it was terminated after 4 weeks of insulin therapy and a 4 week observation period. A second limitation is that, following our observations of the effects of HFD on EIT, the diet may be switched to the LFD at the onset of diabetes in the future study and the total duration of EIT may be assessed. In addition, the impact of HFD on β-cell functions such as glucose-induced insulin secretion (GSIS) at onset of the diabetes and following insulin therapy in both HFD and LFD-treated animals will be evaluated. Lastly, the use of HOMA-IR and HOMA-B% as surrogate measures of insulin resistance and β-cell function is another limitation of this study. These measures correspond well, but are not necessarily equivalent to non-steady state estimates of β-cell function and insulin sensitivity derived from the hyperinsulinemic or hyperglycemic clamp, or the intravenous or oral GTT (0–30 delta Insulin/Glucose). Although a better choice to quantify insulin secretion and sensitivity, clamp studies are difficult to implement. A minor issue was the insulin assay used in this study was not specified to differentiate between the insulin analog and endogenous insulin, but this limitation was mitigated by concomitant measurement of C-peptide levels and the timing of assays.

Nevertheless, our study has established a potential animal model to study the beneficial effect of EIT on glucose control in new onset T2DM. Four weeks of EIT in this HFD-induced T2DM mouse model demonstrated improved GTT performance and a better HOMA-B% following insulin withdrawal at four weeks but only in mice switched to a LFD after insulin treatment. This major finding that improved glycemic control with EIT was only observed and maintained in mice switched to a LFD after insulin therapy may explain why such a similar legacy effect is only achieved in a portion of cases studied, and emphasizes the vital role of diet adherence in diabetes control at any stage of disease progression.

## Supporting Information

S1 Fig Non-fasting blood glucose levels during treatment period. (DOCX)

S2 Fig Blood lipids levels at the end of the experiment. (DOCX)

S3 Fig Oil Red O staining of frozen pancreas sections from mice at the end of the experiment period. (DOCX)

S1 Table Insulin Tolerance Test (ITT) data at the experimental period. (DOCX)

S2 Table Inflammatory markers by Milliplex assay. (DOCX)
