# Supplementary material for: Diet Is Critical for Prolonged Glycemic Control after Short-Term Insulin Treatment in High-Fat Diet-Induced Type 2 Diabetic Male Mice
Source: PLoS One. 2015 Jan 29;10(1):e0117556. doi: 10.1371/journal.pone.0117556 (PMC4310595; doi:10.1371/journal.pone.0117556)
Supplement: S1 Fig — (DOCX) [file pone.0117556.s001.docx]

**Figure S1. Non-fasting blood glucose levels during treatment period**. LS, mice fed a LFD; HS, mice fed a HFD. Both LS and HS groups received Sham treatment. HI, mice fed a HFD and received Insulin treatment.
