# Supplementary material for: Diet Is Critical for Prolonged Glycemic Control after Short-Term Insulin Treatment in High-Fat Diet-Induced Type 2 Diabetic Male Mice
Source: PLoS One. 2015 Jan 29;10(1):e0117556. doi: 10.1371/journal.pone.0117556 (PMC4310595; doi:10.1371/journal.pone.0117556)

**Figure S2. Blood lipids levels.** At the end of the experiment, mice that were kept on the HFD showed high levels of total blood cholesterol levels (***p<0.001, HHI vs. HLI and HHS vs. HLS), whilst triglyceride concentrations were unchanged (c) (n=5-8 per group).


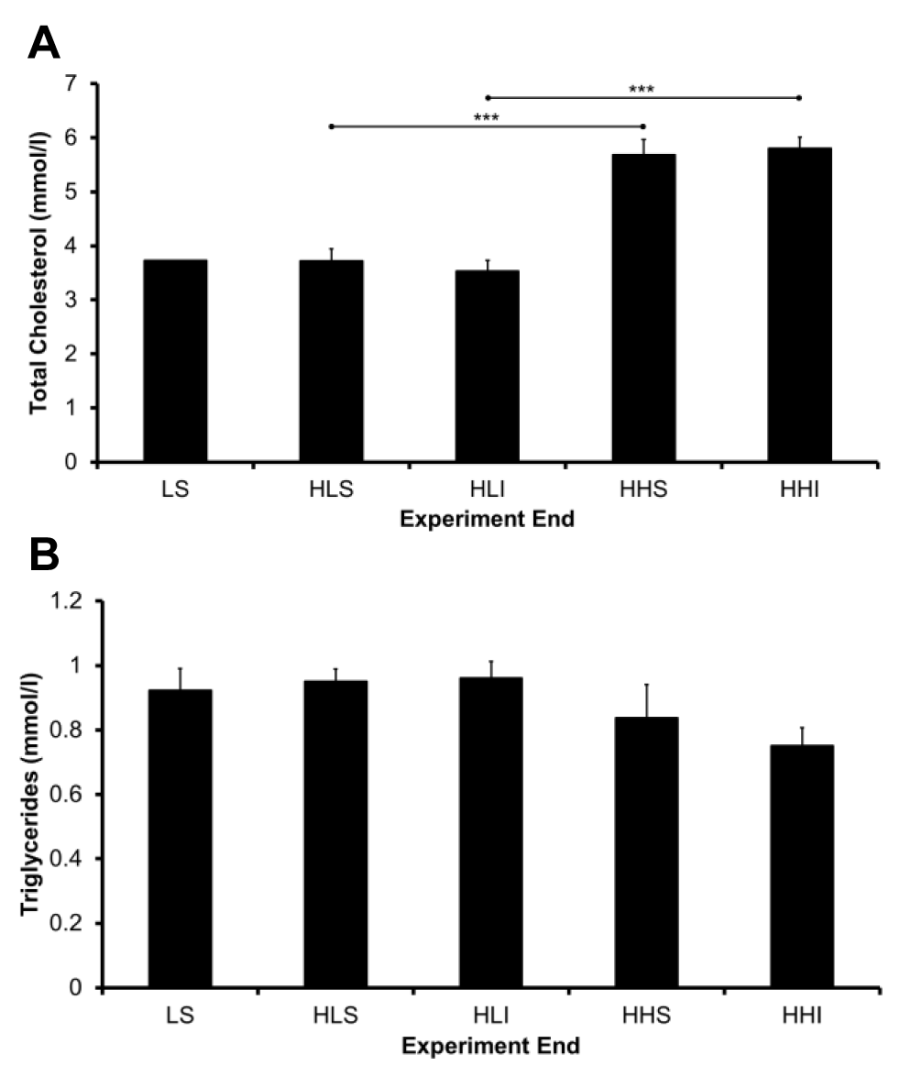

Supplement: S2 Fig — (DOCX) [file pone.0117556.s002.docx]
