# Supplementary material for: Diet Is Critical for Prolonged Glycemic Control after Short-Term Insulin Treatment in High-Fat Diet-Induced Type 2 Diabetic Male Mice
Source: PLoS One. 2015 Jan 29;10(1):e0117556. doi: 10.1371/journal.pone.0117556 (PMC4310595; doi:10.1371/journal.pone.0117556)

**Figure S3.** **Oil Red O staining of frozen pancreas sections from mice at the end of the experiment period.** S3A shows a representative pancreatic islet from an LS mouse. S3B shows a representative pancreatic islet from an HLS mouse. S3C shows a representative pancreatic islet from an HLI mouse. S3D shows a representative pancreatic islet from an HHS mouse. S3E shows a representative pancreatic islet from an HHI mouse. Scale bar = 100 µm.


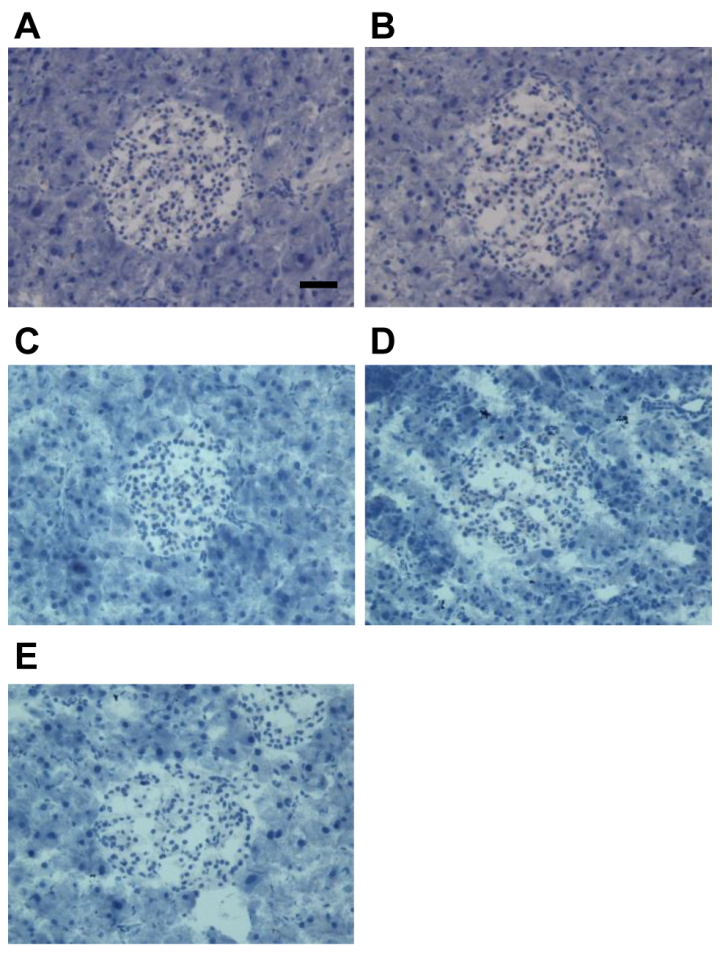

Supplement: S3 Fig — (DOCX) [file pone.0117556.s003.docx]
