# Supplementary material for: Diet Is Critical for Prolonged Glycemic Control after Short-Term Insulin Treatment in High-Fat Diet-Induced Type 2 Diabetic Male Mice
Source: PLoS One. 2015 Jan 29;10(1):e0117556. doi: 10.1371/journal.pone.0117556 (PMC4310595; doi:10.1371/journal.pone.0117556)
Supplement: S1 Table — (DOCX) [file pone.0117556.s004.docx]

**Table S1. Insulin Tolerance Test (ITT) data at the experimental period.**

| **Group (n)** | **0 mins** | **15 mins** | **30 mins** | **45 mins** | **60 mins** | **90 mins** |
| --- | --- | --- | --- | --- | --- | --- |
| **LS (8)** | 10.77 ± 0.44 | 10.76 ± 0.53 | 9.70 ± 1.07 | 9.16 ± 0.68 | 9.45 ± 0.81 | 9.21 ± 0.54 |
| **HLS (8)** | 10.62 ± 0.50 | 9.98 ± 0.46 | 10.48 ± 0.69 | 9.59 ± 0.52 | 10.48 ± 0.38 | 9.87 ± 0.25 |
| **HLI (8)** | 9.13 ± 0.41 | 10.27 ± 0.68 | 8.81 ± 0.55 | 8.57 ± 0.26 | 9.65 ± 0.25 | 9.21 ± 0.33 |
| **HHS (8)** | 13.29 ± 0.90 | 11.34 ± 0.87 | 10.99 ± 0.98 | 10.84 ± 0.79 | 11.43 ± 0.77 | 11.11 ± 0.85 |
| **HHI (8)** | 11.50 ± 0.58 | 9.71 ± 0.42 | 9.64 ± 0.67 | 9.10 ± 0.56 | 10.21 ± 0.66 | 10.48 ± 0.71 |
