# Supplementary material for: Diet Is Critical for Prolonged Glycemic Control after Short-Term Insulin Treatment in High-Fat Diet-Induced Type 2 Diabetic Male Mice
Source: PLoS One. 2015 Jan 29;10(1):e0117556. doi: 10.1371/journal.pone.0117556 (PMC4310595; doi:10.1371/journal.pone.0117556)
Supplement: S2 Table — (DOCX) [file pone.0117556.s005.docx]

**Table S2. Inflammatory markers by Milliplex assay.** LS, mice fed a LFD; HS, mice fed a HFD. Both LS and HS groups received Sham treatment. HI, mice fed a HFD and received Insulin treatment.

| **Group** | **Before Treatment** | | **After Treatment** | | **Experiment End** | |
| --- | --- | --- | --- | --- | --- | --- |
|  | IL-6 | MCP-1 | IL-6 | MCP-1 | IL-6 | MCP-1 |
| LS | 67.33 ± 7.64 | 97.76 ± 28.06 | 7.26 ± 1.05 | Low | 40.40 ± 14.23 | Low |
| HS | 73.37 ± 34.92 | 96.92 ± 27.95 | 18.56 ± 3.20 | 62.75 ± 19.35 | 8.80 ± 3.09 | 93.77 |
| HI |  |  | 39.40 ± 12.05 | 88.23 ± 30.58 | 23.85 ± 5.85 | 158.52 ± 129.23 |
